# Effects of an Oil-Free Hydroethanolic Pumpkin Seed Extract on Symptom Frequency and Severity in Men with Benign Prostatic Hyperplasia: A Pilot Study in Humans

**DOI:** 10.1089/jmf.2018.0106

**Published:** 2019-06-07

**Authors:** Martin Leibbrand, Simone Siefer, Christiane Schön, Tania Perrinjaquet-Moccetti, Albert Kompek, Anca Csernich, Franz Bucar, Matthias Heinrich Kreuter

**Affiliations:** ^1^ Medical Office of Dr. Martin Leibbrand (Urologist), Esslingen, Germany.; ^2^ BioTeSys GmbH, Esslingen, Germany.; ^3^ Frutarom Switzerland Ltd., Scientific Affairs BU Health, Wädenswil, Switzerland.; ^4^ Apomedica Pharmazeutische Produkte GmbH, Graz, Austria.; ^5^ Institute of Pharmaceutical Sciences, University of Graz, Graz, Austria.

**Keywords:** *benign prostatic hyperplasia*, **Cucurbita pepo *L***., *International Prostate Symptom Score*, *lower urinary tract symptoms*, *nocturia*, *pumpkin seeds*

## Abstract

Majority of men are affected by symptomatic benign prostatic hyperplasia (BPH) from a certain age. Botanical extracts are frequently used in the early management of the symptoms. In a single-arm, mono-center pilot study, the effects of a proprietary oil-free hydroethanolic pumpkin seed extract on the symptoms of BPH were investigated. A total of 60 men (62.3 years [95% confidence interval (CI): 60.3–64.3 years]) with a total International Prostate Symptom Score (IPSS) of 14.8 (95% CI: 13.5–16.1) participated between January 2017 and October 2017 in the study by ingesting the oil-free hydroethanolic pumpkin seed extract once daily before going to bed during 3 months. Change in IPSS within treatment period was assessed. Frequency of nocturia was recorded by bladder diary, and postvoid residual urine volume was determined through ultrasound. Between baseline and after 12 weeks of supplementation, a significant symptom reduction of an average 30.1% (95% CI: 23.1–37.1) was seen for the total IPSS. Symptom alleviation had a high impact on quality of life (*P* < .0001) and was significant after 8 and 12 weeks of intervention (*P* < .001). Nocturia significantly decreased over time (*P* < .0001), as confirmed by IPSS questionnaire and bladder diary. Postvoid residual urine volume was significantly reduced at the end of intervention (baseline: 83.67 mL [95% CI: 58.02–109.3]; after 12 weeks: 63.11 mL [95% CI: 45.37–80.85]; *P* = .0394). These results indicate that the oil-free hydroethanolic pumpkin seed extract seems to be a very well tolerable, appropriate plant extract to support health benefits in a collective suffering from BPH related symptoms without the need of medical treatment.

## Introduction

Benign prostatic hyperplasia (BPH) is an age-related condition characterized by histological changes of the prostate stromal and glandular tissue showing increased proliferation rate around the urethra, resulting in prostatic enlargement and increased stromal smooth muscle tone. Eventually, bothersome lower urinary tract symptoms (LUTS) occur, which can have a significant impact on life quality. A common measure of LUTS is the validated International Prostate Symptom Score (IPSS), a questionnaire developed by the American Urological Association.^4,5^

The prevalence of histological diagnosis is high; however, not all men with confirmed alterations are affected: about half of those 80 years and older report urinary symptoms.^1^ BPH is very common during aging and is first detectable around the fourth decade, with prevalence increasing with age. Every second man between 51 and 60 shows histological BPH, while almost all men are affected in the ninth decade.^2,3^

Clinical LUTS include nocturia, frequent micturition, urge symptoms, urinary flow weakening, or residual urine formation and are progressing with age. When the prostatic tissue enlargement develops to compress the urethra and results in physical bladder outlet obstruction, the resulting symptoms become troublesome.^1,6^ Furthermore, the overstimulation of smooth muscle tone through *α*-adrenergic receptors can worsen symptoms of obstruction, resulting in retention of urine and decreasing the urinary flow rate.^6^ Commonly drug classes used for BPH treatment (*α*-antagonists, 5-*α*-reductase inhibitors) focus on reversing these mechanisms.

For those men presenting with mild-to-moderate symptoms but limited discomfort due to their symptoms, watchful waiting (*i.e.*, a strategy of yearly reevaluation) and reassurance are suggested as appropriate procedures.^1^ This strategy is based on the observation that progression of symptoms in these patients is rare and that development of serious complications is uncommon. In this phase, the use of botanicals to support voiding function is of great interest. In this context, there is some evidence and traditional recognition for pumpkin seed extract.^7–9^

In a narrative review by Damiano *et al.*^10^ the role of *Cucurbita pepo* of the gourd family (*Cucurbitaceae)* in the management of patients affected by LUTS due to BPH was summarized. In the context of this narrative review, extracts of *C. pepo* seem to show significant efficacy in improving urinary symptoms. The clinical data have been collected with conventional pumpkin seed preparations, including pumpkin seed powders, for example, seeds milled to powder or pumpkin seed oil or a whole-seed extract manufactured from the seeds by extraction with almost pure ethanol. This is in contrast to the proprietary oil-free hydroethanolic pumpkin seed extract (EFLA^®^940; Frutarom Switzerland Ltd., Wädenswil, Switzerland) from pumpkin seeds of *Cucurbita pepo* L. ssp. *pepo* var. *styriaca* (a medicinal variety of pumpkin, the “Styrian oil pumpkin”), which was investigated in the current study.

For this extract only a combination with Soy germ extract was investigated on pollakiuria in the night in elderly men with positive outcome.^11^ Also an activity reduction of prostatic 5-*α*-reductase, with inhibiting effects on prostate tissue growth, was shown *in vitro*, as well as in an animal model (*in vivo*), for this extract.^12^ However, for the health issue BPH there are no clinical data available on the pure extract using validated instruments like IPSS. Due to the different composition of the *C. pepo* extracts described in literature and accompanied clinical data, the data cannot be extrapolated to the oil-free extract. Effects need to be documented for each extract. To overcome these limitations and estimate the effects of the oil-free hydroethanolic pumpkin seed extract, this single-arm pilot study was conducted.

## Materials and Methods

### Study design

The study was conducted as an open mono-center trial with 60 men with symptomatic BPH. The intervention period was 3 months with visits at study site every 4 weeks (four visits, in total). The study was conducted in compliance with International Conference on Harmonization - Good Clinical Practice guidelines and the declaration of Helsinki and was reviewed by the Institutional Review Board “Landesärztekammer Baden Württemberg” without any concerns (F-2016-110). The study was registered in DRKS (German Clinical Trials Register), (DRKS00011044).

The study was performed at the medical office of Dr. Martin Leibbrand (independent urologist) in Esslingen with support by BioTeSys GmbH (Esslingen, Germany), an independent clinical research organization with focus on nutritional research.

### Subjects

**FIG. 1.**
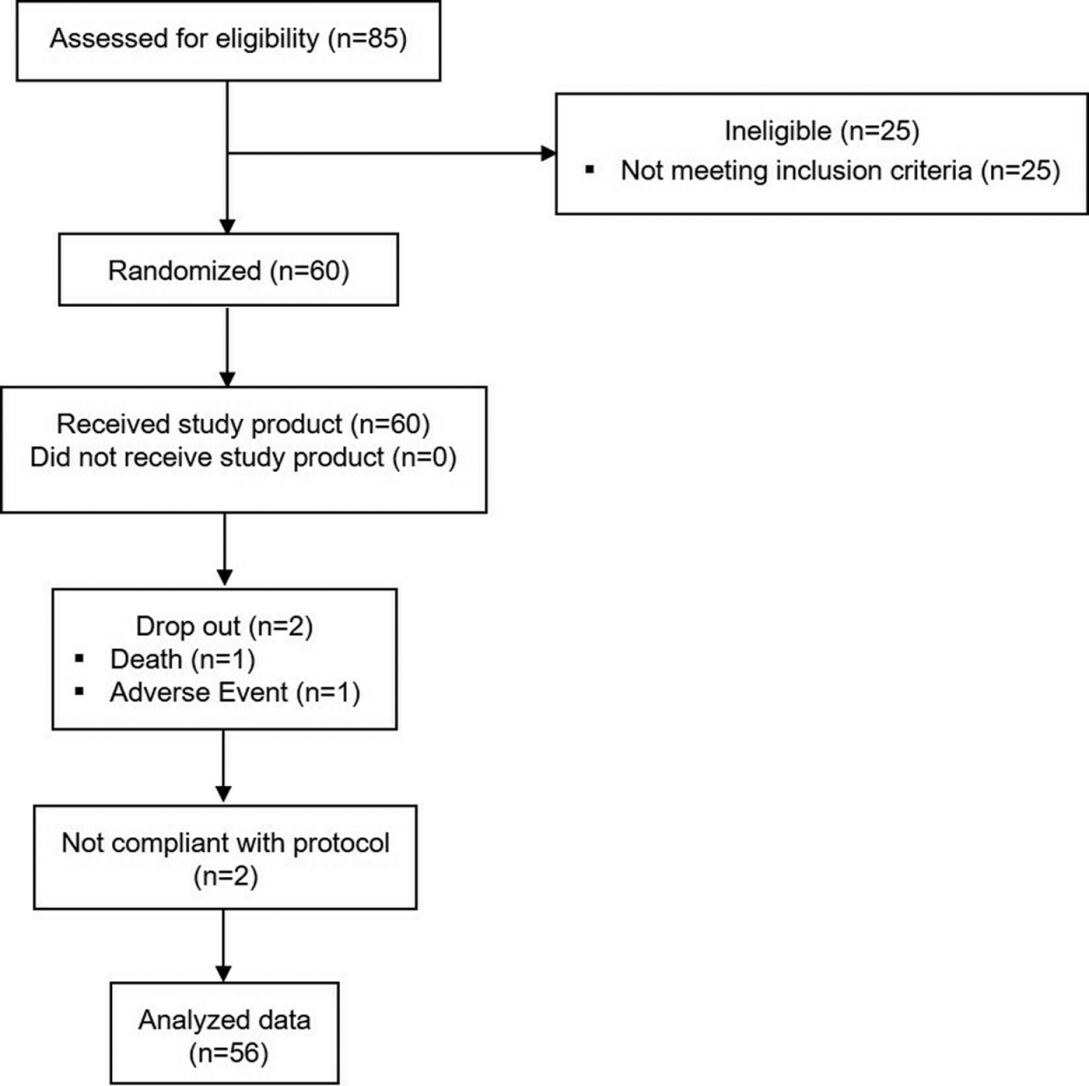
Consort flow diagram.

Figure 1 shows the flow of participants through the study. Recruitment started in January 2017. The last subject finished the study in October 2017. Of the 85 men screened, 60 met inclusion criteria and were enrolled. Fifty-eight successfully finished the study, and *n* = 56 was evaluated in the per protocol analysis, defined as the primary analysis (reasons for drop outs: 1 × serious adverse event, 1 × adverse event [AE]; reason for exclusion from per protocol analysis: 1 × not meeting inclusion criteria at start of intervention [baseline]: IPSS <8 at baseline; 1 × clinical finding during study conduct huge postvoid volume >800 mL confirmed at baseline and at the end of intervention).

Inclusion criteria were as follows: men, aged between 50 and 75 years, suffering from symptoms of BPH for at least 6 months before screening, prostate-specific antigen (PSA) <4 ng/mL, IPSS ≥8 at screening, and nocturia: at least 1.5 per night.

The main exclusion criteria were as follows: history of prostatic surgery (including transurethral resection of the prostate, balloon dilatation, thermotherapy, and/or stent replacement) or other invasive or minimally invasive procedures to treat BPH, other conditions that may cause urinary symptoms (*e.g.*, neurogenic bladder, bladder neck contracture, urethral stricture, bladder malignancy, acute or chronic prostatitis, or acute or chronic urinary tract infections), history or evidence of prostate cancer (*e.g.*, positive biopsy or ultrasound, suspicious digital rectal exam, and/or rising PSA), body mass index ≥40 kg/m^2^, glycated hemoglobin (HbA_1c_) ≥6.5% at screening in nondiabetic subjects, HbA_1c_ ≥7.5% at screening in subjects under medical treatment of diabetes.

Further exclusion criteria were: history of surgery of the prostate, bladder, or urethra, use of any other medications or dietary supplements for treatment of BPH, associated symptoms and erectile dysfunction in the past month, for this study clinically relevant abnormal laboratory, vital signs or physical findings at screening, gastrointestinal diseases/conditions (colitis ulcerosa, Crohn's irritable bowel syndrome, inflammatory bowel disease, peptic ulcers, and celiac disease), and heavy smoker >15 cigarettes per day.

### Test substance

The test substance is a proprietary hydroethanolic extract (EFLA^®^ 940) from pumpkin seeds of *Cucurbita pepo* L. ssp. *pepo* var. *styriaca* (a medicinal variety of pumpkin, the “Styrian oil pumpkin”), defatted by removal of oil through pressing. It is manufactured by extraction with 60% (m/m) ethanol/water followed by a proprietary decontamination procedure (EFLA^®^ HyperPure) for the removal of remaining oil traces, adjusted with additives and dried. It is characterized by a drug:extract ratio of 15–25:1 (DER native 21–36:1) (by weight), related to the fresh pumpkin seeds. The extract was pressed in tablets only adding commonly used excipients but no other active ingredients. Tablets were provided by Apomedica Pharmazeutische Produkte GmbH (Dr. Böhm^®^ Kürbis). The volunteers ingested one tablet daily (500 mg of the extract, corresponding to 350 mg native extract and equivalent to 10 g of pumpkin seeds per tablet) before going to bed.

Compliance was determined by counting residual returned tablets and considered sufficient if >80% of the correct quantity of the investigational product were consumed. The extract is high in phenolic content with 3.2% (m/m) phenolic derivatives based on the colorimetric Folin–Ciocalteau method and determined as enterodiol using a conversion factor of 2.4 for pyrogallol to enterodiol in the calculation. Furthermore, the extract was characterized by the following components 0.219% (m/m) adenosine and 0.63% (m/m) cucurbitin, which are natural ingredients of the pumpkin seeds. The tablets only contained commonly used excipients but no other active ingredients besides EFLA 940 pumpkin seed extract.

### Data collection

The IPSS, a 8 question (7 symptom questions +1 quality of life [QoL] question) validated screening tool used to track the symptoms of the BPH, was completed at screening and at each study visit.^4,5^ Each question concerning urinary symptoms allows the patient to choose one out of six answers (scale 0–5: question 1–6: 0 = not at all to 5 = always; question 7 [nocturia]: 0 = never up to 5 = ≥5 times) indicating increasing severity of the particular symptom.

An IPSS total score of 0–7 is classified as mild symptoms. Moderate symptoms are defined with a score of 8–19, and 20–35 score points are classified as severe symptoms. Only the subjects with IPSS ≥8 were included in the study. Furthermore, 1 week before the visits, the subjects were instructed to document each voiding of urine and indicate day and night time in a bladder diary. Average frequency of voiding during day and night was calculated based on the diaries. Residual postvoid urine volume was determined using medical ultrasound at the beginning and at end of supplementation.

As safety parameters, hemogram, lipid status, HbA_1c_ (only screening), and PSA level (chemiluminescence immunoassay technique) were determined at screening visit and at end of supplementation after at least 3 h of fasting by an accredited routine laboratory (Synlab Laboratories, Leinfelden). Furthermore, concomitant medications and AEs were monitored.

### Statistics and data analysis

Changes over time were evaluated as exploratory. Statistical analyses were performed with GraphPad Prism version 5.04. All statistical tests were performed two sided. A significance level of 0.05 was used. Non-normality was evaluated with Shapiro–Wilk test (*α* level of 0.1). As efficacy parameters were non-normally distributed, datasets with four assessment time points (*i.e.*, IPSS, frequency of micturitions, and so on) were evaluated with Friedman test and Dunn's multiple comparison test as post-test comparing baseline assessment with the 4, 8, and 12 weeks of intervention assessment. Responder rate of subjects with IPSS reduction >4 score points was additionally estimated. From literature placebo reductions of up to 4 score points were estimated within 3 months of intervention.

In case of only two assessment time points (baseline and end of intervention, *e.g.*, postvoid volume), Wilcoxon signed rank test was applied. For evaluation of postvoid residual volume, subjects were additionally classified according to their postvoid volume at baseline (<50 mL, 50–100 mL, and >100 mL). If not specified otherwise, mean values with 95% confidence interval (CI) are reported.

## Results

### Demographic and baseline characteristics

Baseline characteristics for the 56 subjects finishing the study according to protocol are depicted in Table 1. All subjects showed a normal PSA level, with the limit for inclusion for PSA defined at <4 ng/dL. Furthermore, all subjects showed an HbA_1c_ level of <6.5% for nondiabetics and for diabetic subjects <7.5%, respectively. Overall, three diagnosed diabetics participated in the study. During screening visit the subjects were asked for their medical history and regular intake of medication. None of the medication was in conflict with the study procedures.

**Table 1.** Demographic and Baseline Characteristics

*Variable*	*Mean (95% CI)*
Age (years)	62.3 (60.3–64.3)
BMI (kg/m^2^)	26.3 (25.3–27.3)
SBP/DBP (mmHg)	149.8 (143.6–154.1)/89.6 (86.6–92.7)
HbA_1c_ (%)	5.5 (5.4–5.6)
PSA (ng/mL)	1.3 (1.0–1.5)
IPSS (score)	14.8 (13.5–16.1)

CI, confidence interval; DBP, diastolic blood pressure; BMI, body mass index; IPSS, International Prostate Symptom Score; PSA, prostate-specific antigen; SBP, systolic blood pressure.

All subjects were in a stable state before and during the conduct of the study. Most frequent chronic disorders reported were elevated blood pressure or elevated blood lipids followed by diseases referring to the musculoskeletal system like arthrosis. Only the subjects with at least moderate symptoms of the urinary tract system but no need of medical treatment were enrolled in the study (IPSS ≥8). On average, the IPSS was 14.8 (95% CI: 13.5–16.1).

The overall compliance of the study product intake was excellent with 100.6%. The compliance was calculated from returned study products. The value >100% can be explained by unintended double intake or loss of products. No subject met the noncompliance criterion of <80%.

### International Prostate Symptom Score

**FIG. 2.**
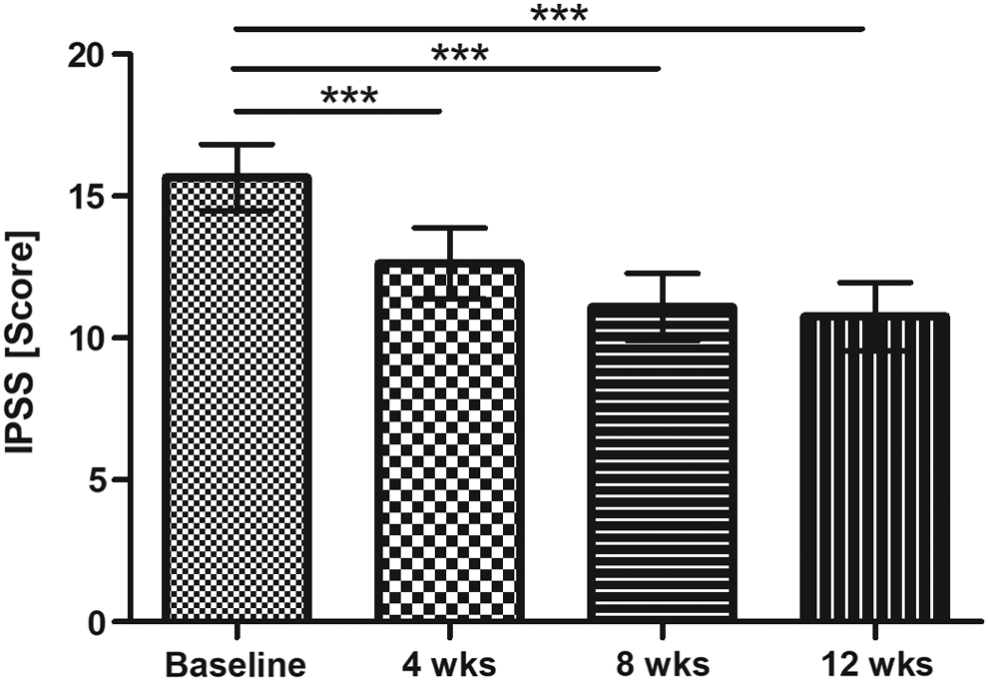
IPSS (score) (mean ±95% CI; *P* < .0001 Friedman test; results of Dunn's multiple comparison test are presented ***P < .001). CI, confidence interval; IPSS, International Prostate Symptom Score.

At baseline, 43 out of 56 subjects (76.8%) showed moderate symptoms and 13 subjects (23.2%) severe symptoms. During intervention with the oil-free hydroethanolic pumpkin seed extract, there was a highly significant reduction of symptoms after 12 weeks of supplementation (baseline: 15.7 [95% CI: 14.5–16.8]; after 12 weeks: 10.8 [95% CI: 9.5–12.0]; *P* < .0001). The symptom alleviation was already significant after 4 weeks of intervention (*P* < .001) and strengthened over the whole study period with significant reductions of IPSS after 8 and 12 weeks (*P* < .001) (Fig. 2). At the end of the intervention, in 20 subjects (35.7%) the symptoms could be reduced to mild symptoms (IPSS <8), and only 1 of the subjects remained in the category with severe symptoms.

On average, a symptom reduction over time was seen during the supplementation phase between baseline and after 4, 8, and 12 weeks (17.1% [95% CI: 10.0–24.2] after 4 weeks, 27.5% [95% CI: 20.7–34.4] after 8 weeks, and 30.1% [95% CI: 23.1–37.1] after 12 weeks), respectively.

A symptom alleviation greater than 4 score points was achieved by 55.4% (31 subjects) of the participants. When comparing baseline and 12 weeks of supplementation 12.5% (7 subjects) experienced slight worsening of symptoms, 3.6% (2 subjects) did not show any change, and 28.6% (16 subjects) showed a reduction between 1 and 4 score points.

**FIG. 3.**
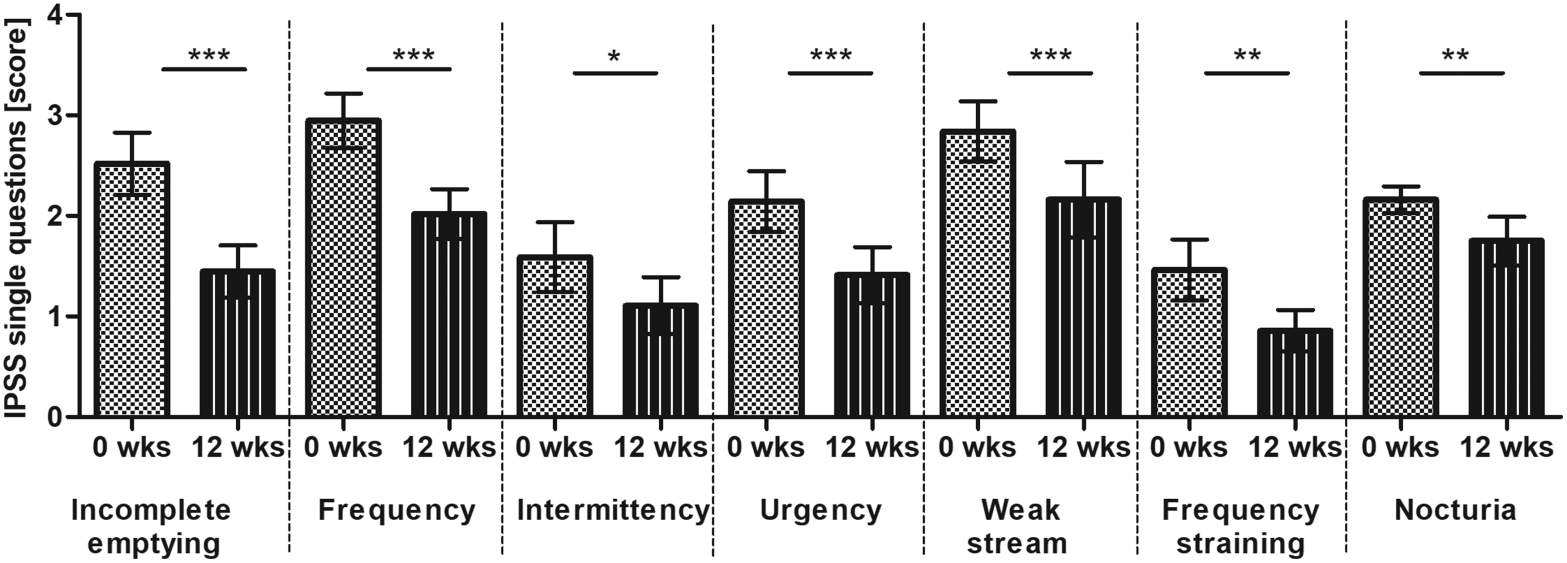
IPSS of single questions (score) (mean ±95% CI; results of Friedman test are presented in Table 2; Dunn's multiple comparison test between baseline (week 0) and week 12 is presented *P < .05; **P < .01; ***P < .001). Score 0: not at all, score 1: less than 1 in 5 times; score 2: less than half the time; score 3: about half the time, score 4: more than half the time; score 5: almost always.

The data show that for every BPH related symptom assessed with IPSS questions 1–7, respectively, a significant symptom reduction was reported over time, see Table 2 and Figure 3. Greatest effects were seen for the end points “incomplete emptying” and “frequency of micturitions with less than every two hours” for which the scores changed on average by 1 score point. For incomplete emptying the score changed from 2.5 (95% CI: 2.2–2.8) to 1.5 (95% CI: 1.5–1.7) equivalent to the category in-between “about half the time (score 3)” and “less than half the time (score 2)” to in-between “less than half the time (score 2)” and “less than 1 in 5 times (score 1).” The “frequency” could be improved on average from category “less than half the time” to “less than 1 in 5 times.”

**Table 2.** Descriptive Statistics of Single Questions 1–7 of International Prostate Symptom Score at Baseline and 4, 8, and 12 Weeks of Intervention with Oil-Free Hydroethanolic Extract from Pumpkin Seeds of *Cucurbita pepo* (Score)

*IPSS (single questions)*	*Baseline*	*4 Weeks*	*8 Weeks*	*12 Weeks*	P
Question 1: Incomplete emptying	2.5 (2.2–2.8)	1.9 (1.6–2.2)	1.7 (1.4–2.0)	1.5 (1.2–1.7)	<.0001
Question 2: Frequency	3.0 (2.7–3.2)	2.3 (2.1–2.6)	2.0 (1.8–2.3)	2.0 (1.8–2.3)	<.0001
Question 3: Intermittency	1.6 (1.2–1.9)	1.4 (1.1–1.8)	1.3 (0.9–1.6)	1.1 (0.8–1.4)	.0074
Question 4: Urgency	2.1 (1.8–2.4)	1.5 (1.3–1.8)	1.5 (1.2–1.7)	1.4 (1.1–1.7)	<.0001
Question 5: Weak stream	2.8 (2.5–3.1)	2.4 (2.0–2.7)	2.1 (1.8–2.5)	2.2 (1.8–2.5)	<.0001
Question 6: Frequency straining	1.5 (1.2–1.8)	1.1 (0.8–1.4)	0.8 (0.6–1.1)	0.9 (0.7–1.1)	.0001
Question 7: Nocturia	2.2 (2.0–2.3)	2.0 (1.8–2.3)	1.7 (1.6–1.9)	1.8 (1.5–2.0)	<.0001

### IPSS quality of life

The symptom alleviation had also a significant impact on QoL (*P* < .0001), which was significant after 8 and 12 weeks of intervention (*P* < .001). During the supplementation with the hydroethanolic pumpkin seed extract the percentage of the subjects being “pleased” with QoL could be greatly increased from 5.4% (3 subjects) at baseline to 35.7% (20 subjects) at the end of the supplementation. In parallel, the percentage of the subjects being unhappy at the beginning with 21.4% (12 subjects) at baseline could greatly be reduced to 3.6% (2 subjects) at the end of the intervention, see Table 3 (IPSS-QoL). At the end of the intervention, 60.7% (34 subjects) of the subjects reported an improvement on QoL when comparing with baseline judgment. In 32.1% (18 subjects) of the cases no change of QoL was reported, and in rare cases of 7.1% (4 subjects) even a worsening of QoL was reported.

**Table 3.** Frequency [% (*n*)] with Categories of International Prostate Symptom Score-Quality of Life at Baseline and 4, 8, and 12 Weeks of Intervention with Oil-Free Hydroethanolic Extract from Pumpkin Seeds of *Cucurbita pepo*

*IPSS-QoL response categories [% (n)]*	*Baseline*	*4 Weeks*	*8 Weeks*	*12 Weeks*
Delighted	0 (0)	3.6 (2)	0 (0)	1.8 (1)
Pleased	5.4 (3)	14.3 (8)	28.6 (16)	35.7 (20)
Mostly satisfied	28.6 (16)	35.7 (20)	41.1 (23)	33.9 (19)
Mixed	44.6 (25)	32.1 (18)	21.4 (12)	23.2 (13)
Unhappy	21.4 (12)	8.9 (5)	7.1 (4)	3.6 (2)
Terrible	0 (0)	5.4 (3)	1.8 (1)	1.8 (1)

QoL, quality of life.

### Frequency of micturition during day and night

**FIG. 4.**
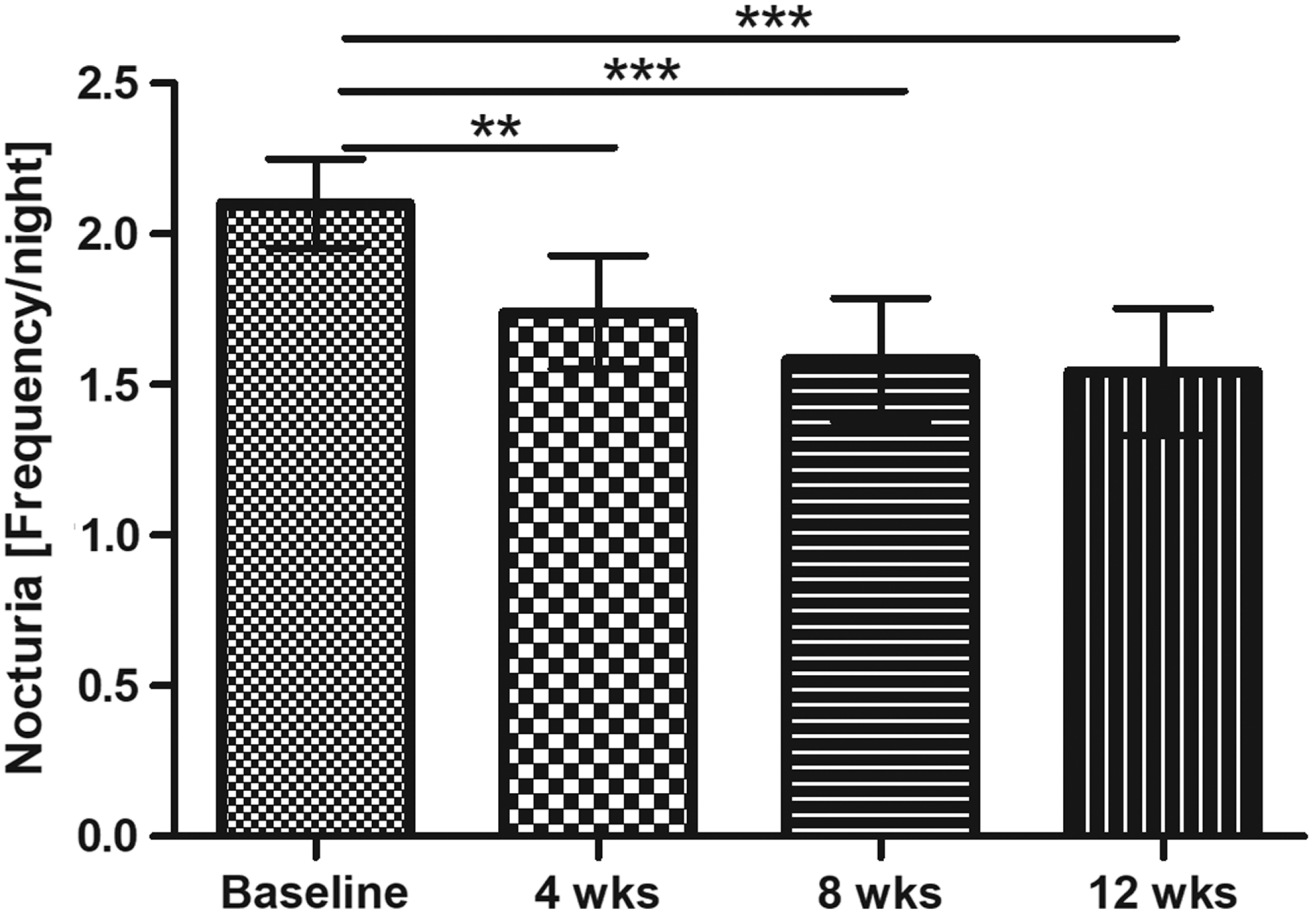
Nocturia (frequency/night) (mean ±95% CI; *P* < .0001 Friedman test; results of Dunn's multiple comparison test are presented ***P < .001, **P < .01).

Before each visit, micturitions were documented prospectively in a diary over a period of 1 week. Overall a significant reduction of total micturitions was seen (*P* = .0121). The significant reduction was confirmed by the pairwise tests in comparison to baseline for the 8- and 12-week assessment (*P* < .05). Investigating the frequency of micturitions during daytime and during the night separately, data indicate that the improvement was obvious in the night, but not during day time (*P* = .5461). During night, there was a highly significant reduction of nocturia over time (*P* < .0001). This decrease had already significantly developed after 4 weeks of intervention with the oil-free hydroethanolic pumpkin seed extract (1 tablet per day of EFLA 940, no additional bioactive ingredients) and nocturia continued to decline until the end of the study, see Figure 4.

After 12 weeks of intervention, nocturia could be reduced by an average of 0.56 micturitions per night (95% CI: 0.38–0.74). Possible confounding factors on nocturia were controlled. During the weeks of recordings before the visits, the average duration of nights was comparable with around 8 h (*P* = .6461). Furthermore, the subjects estimated their drinking volume each day. There was no change of average drinking amount (*P* = .8246) over the study period. On average, the subjects reported to drink slightly more than 2 L per day.

### Postvoid volume

**FIG. 5.**
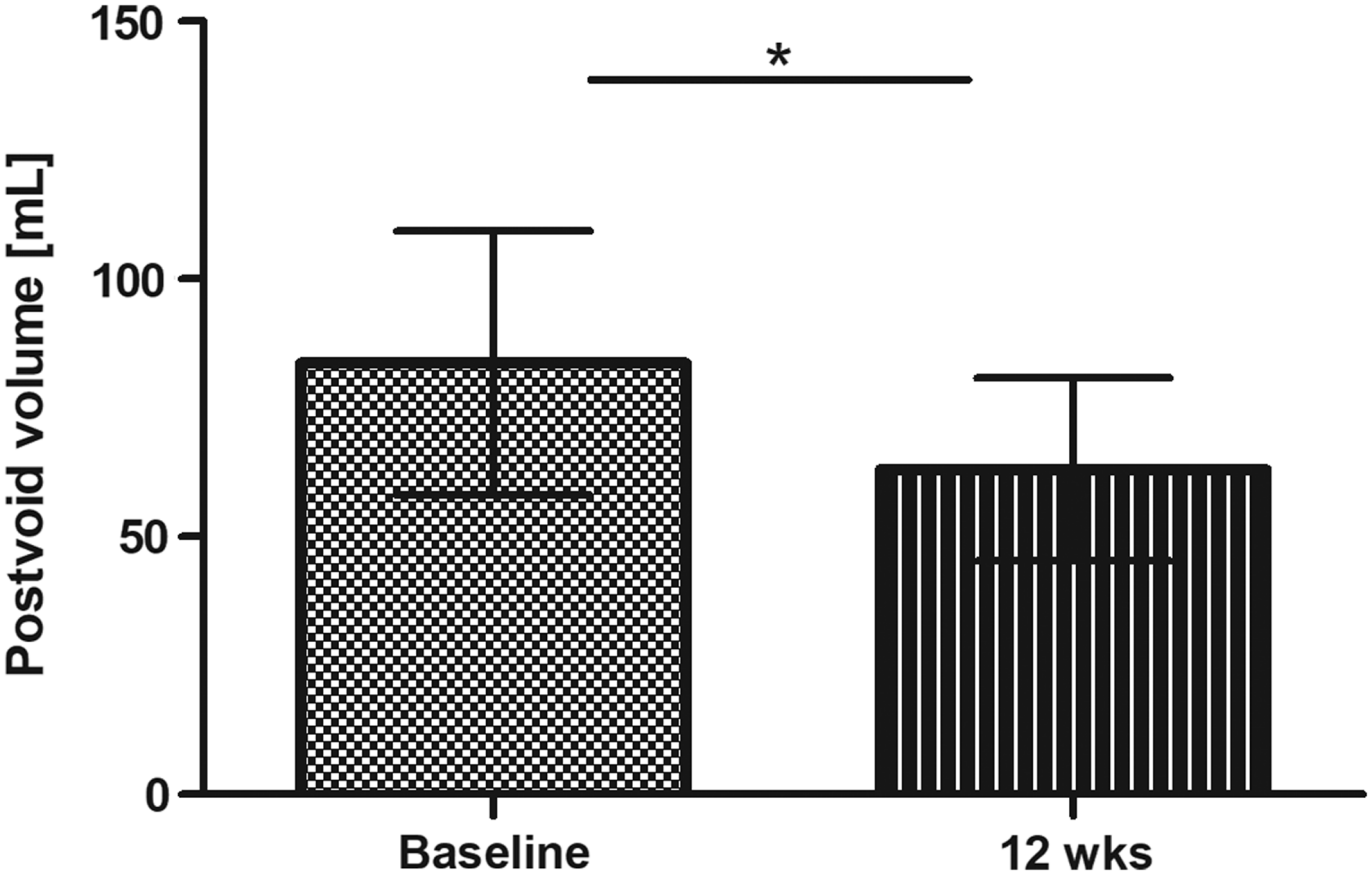
Postvoid volume assessed with ultrasound at baseline and end of intervention (mean ±95% CI; *P < .05).

Data indicate a significant reduction of postvoid urinary volume (baseline: 83.67 mL [95% CI: 58.02–109.3]; end of intervention: 63.11 mL [95% CI: 45.37–80.85]; *P* = .0394), see Figure 5.

According to the categorical evaluation, 51.8% of the subjects (*n* = 29) showed postvoid volume <50 mL, 23.2% (*n* = 13) a postvoid volume of 50–100 mL, and 25% (*n* = 14) a postvoid volume >100 mL at the beginning of the study. From the 29 subjects with postvoid volume <50 mL at baseline, 72.4% (21 men) remained in this category, whereas 17.2% (5 men) showed slightly elevated amounts with 50–100 mL and 10.3% (3 men) showed a postvoid volume >100 mL at the end of intervention.

From the 13 subjects in the 50–100 mL category at baseline, only 1 remained in this category, 10 improved to a postvoid volume <50 mL, and only 2 showed a postvoid volume >100 mL at the end of intervention. Overall, in these subgroups postvoid volume was reduced by trend (baseline: 69 mL [95% CI: 60–78]; end of intervention: 44 mL [95% CI: 12–76]; *P* = .0681).

From the 14 subjects with a postvoid urine volume >100 mL at the beginning of the study, 11 subjects (78.6%) remained in this category, whereas 2 subjects improved to volumes <50 mL and 1 subject ended with 50–100 mL postvoid volume. Although the majority of the subjects remained in the highest category of the postvoid volume, the postvoid volume was significantly reduced in this subgroup (baseline: 228 mL [95% CI: 180–275]; end of intervention: 130 mL [95% CI: 95–164]; *P* = .0023).

### Global assessment

The majority of subjects (78.6%) reported an overall reduction in BPH associated symptoms at the end of the 12-week intervention. The subjects indicating an improvement explained the improvement predominantly by less nocturia, overall less frequency of voiding, and less urgency.

### Safety

During intervention phase 66 AEs were reported by 29 subjects. None of these AEs was related to the study products. Two of these AEs were classified as serious due to death of one subject and due to hospitalization of a second subject, respectively. Overall, headache and diseases of the respiratory tract like upper respiratory tract infections, followed by diseases like knee and back pain, were the most commonly reported AEs.

The PSA level was measured at screening and at the end of the study. Only subjects with PSA levels <4 ng/mL were included. A slight but significant increase was measured at the study end (baseline: 1.25 ng/mL [95% CI: 1.04–1.45]; end of intervention: 1.52 ng/mL [95% CI: 1.22–1.82]; *P* = .0057), whereby all PSA levels except for three subjects (1.51 up to 5.93 ng/mL, 3.42 up to 4.92 ng/mL, and 0.54 up to 4.0 ng/mL) were still <4 ng/mL.

## Discussion

The BPH is the most common neoplastic condition afflicting men and constitutes a major factor impacting health. Most patients with medical problems caused by BPH present symptoms of dysfunctional voiding. This symptom complex is nonspecific and includes incomplete emptying, weak flow, nocturia, etc.

The prevalence of histological diagnosed prostatic hyperplasia increases from 8% in men aged 31–40 years, to 40–50% in men aged 51–60 years, and to over 80% in men older than 80 years.^13^ The histological BPH, although identified by the International Classification of Diseases code 600, does not necessarily constitute a problem for the patient. The condition becomes a clinical entity when it is associated with subjective symptoms, the most common manifestation being LUTS.^14^ The often bothersome irritative symptoms consist of frequency, urgency with urge incontinence, nocturia, and painful urination, as well as small voided volumes. For the current clinical study the selection of the subjects was based on LUTS assessed using IPSS and nocturia.

A 12-week intervention with the proprietary oil-free hydroethanolic pumpkin seed extract demonstrated a significant benefit on symptomatic BPH as assessed with IPSS questionnaire, bladder diary, and postvoid volume.

On average, a reduction of IPSS of 30.1% was seen between baseline and end of intervention. The findings are in line with other investigations with another pumpkin seed extract. Friederich *et al.*^15^ reported a symptom alleviation of IPSS from 18.64 to 10.94 after 3 months of intervention in a huge observational study with *n* = 2254 subjects. Bach^16^ reported a decrease of IPSS from 17.6 to 10.9 after 12 months of intervention.

However, in this study also a placebo effect was observed with a reduction from IPSS 17.7 to IPSS 12.2, thus an IPSS response up to 5 points within 12 months might be attributed to placebo effects. After 3 months possible placebo effects were estimated with an improvement of up to 4 points. Therefore, in our trial the rate of responder was calculated based on this cutoff (reduction >4 score points). According to this definition, 55.4% of participants could be classified as responders. In the study of Vahlensieck *et al.*^17^ the primary outcome was a decrease in IPSS of >5 points from baseline after 12 months. The response rate differed significantly between pumpkin seed and placebo (58% vs. 47%). The response rate is therefore comparable to the response rate seen in our study taking into account the shorter intervention time and the adjusted cutoff level for responder definition.

Investigating the single questions of IPSS indicated that there was a significant improvement for all assessed symptoms during intervention period. The symptom alleviation showed also a significant benefit on QoL. A significant improvement of QoL was also reported by Friederich *et al.*^15^ and Bach.^16^

Overall, there was a significant reduction of micturitions, which was most pronounced during the night (*P* < .0001). On average after 12 weeks of intervention, nocturia could be reduced by 0.56 micturitions per night. The prospectively assessed data in the bladder diary are in line with the retrospectively assessed IPSS question No. 7 confirming a reduction by 0.41 micturitions per night. Anyhow, data indicate that nocturia is reflected more thoroughly by the 1-week bladder diaries taking into account the normal physiologic variability, which might explain the slight underestimation in the retrospective assessment with the IPSS questionnaire. In the study of Friederich *et al.*^15^ nocturia was evaluated through question No. 7 of the IPSS questionnaire. In this observational study, a reduction from 2.7 to 1.3 is reported. In contrast to these results, no reduction of nocturia was reported in the placebo-controlled study of Bach.^16^ In this study, only a reduction of micturitions during the day was reported.

The frequent micturitions and urinary incontinence are discussed to be caused by a hormonal imbalance between androgens and estrogens.^18^ This is accompanied with an overproduction of 5-*α*-reductase. An inhibition of 5-*α*-reductase, with inhibitory effects on prostate tissue growth, was shown *in vitro*, as well as in an animal model (*in vivo*), for the extract EFLA 940.^12^ As further mode of action, it could be shown that the extract inhibits the activity of the enzyme aromatase *in vitro* and, thus, results in increase of testosterone levels. These results indicate that the inhibition of 5-*α*-reductase and aromatase might be an important mode of action and needs further investigation.^12^

In a narrative review of Damiano *et al.*^10^ further *in vitro* mode of actions of *C. pepo* extracts like antioxidative and anti-inflammatory activity were summarized.^19^ Further animal studies also confirmed the inhibitory effects on prostate tissue growth in animal models with different *C. pepo* extracts.^20,21^ Our data found with the nonoily extract of *C. pepo* support that the effects on clinical symptoms are caused by semipolar or water soluble polar substances present in the oil-free pumpkin seed extract. Further research is necessary to identify the bioactive ingredients.

Incomplete retention is diagnosed by a postvoid residual volume >100 mL in patients older than 65 years.^22^ But also cutoff values of <50 mL are reported in literature as a norm value for healthy subjects.^23^ Further data evaluation by categories according to postvoid volume at baseline data indicate that subjects with a postvoid volume >50 mL benefit most from the intervention with the oil-free hydroethanolic pumpkin seed extract seen as a reduction by trend (*P* = .0681) in the intermediate group and a significant reduction in subjects starting with postvoid urine volume >100 mL (*P* = .0023).

In a study of Jeyaraman and Patki^24^ investigating a polyherbal formulation in BPH patients over a period of 2 months, postvoid residual volume was also measured and a significant reduction was reported from 85.3 to 52.1 mL. The difference was highly significant in comparison to placebo. In the placebo group no difference was reported over the study period. It seems that the postvoid measure shows less impact of placebo effects in comparison to subjective assessment tools like the IPSS. The extent of postvoid reduction is in line with the extent found in our study. Comparable effects for a *C. pepo* preparation were reported in the study of Hamvas *et al.*^25^ with a decrease of postvoid residual volume from 90 mL (baseline) to 50 mL (after 10 months of intervention).

In an animal study, urinary bladder function was investigated through cystometrogram using anesthetized rats. The hydroethanolic extract from oil-free pumpkin seeds EFLA 940, which was also used in the current study, induced a significant decrease of excretion frequency and a significant increase of the excretion delay index.^26^ These data underline the observations reported for postvoid volume in the current study and support the health benefits.

No benefit on PSA levels were seen during the intervention with the oil-free hydroethanolic pumpkin seed extract. There was even a slight but significant increase. One explanation for this overall minor slight increase might be increasing physical activity as a result of seasonal variations, as the study start was in winter and the end in late spring or summer. Physical activity increases with increasing outdoor temperatures, and more outdoor activities like gardening, cycling, or hiking were done by subjects. No relation to the study product intake could be seen by the investigator. Furthermore, in literature, different external influences are discussed to greatly impact the PSA levels (*e.g.*, sexual activity, cycling or rectal manipulations as colonoscopy, prostate massage, or urinary retention) during the days before blood sampling.^27,28^ These possibly confounding factors were not controlled during the conduct of the study, so it is unknown if one or more of these interacting factors were applicable to the subjects.

One limitation of the study is the open label single arm design which did not control for placebo effects. It is known that symptoms of BPH are prone to placebo effects. However, considering possible placebo effects estimated from literature, the benefit of the oil-free pumpkin extract could still be demonstrated with the responder analysis. Furthermore, there was also a positive effect on objective measures of BPH symptoms (*e.g.*, postvoid volume) which also demonstrated a health benefit for EFLA 940, the oil-free hydroethanolic pumpkin seed extract. Overall the very good consistency of study results between the different assessment tools strengthens the findings. Anyhow, results should be confirmed in a placebo controlled double blind study.

## Conclusion

The aim of the current study was to investigate the effects of an oil-free hydroethanolic pumpkin seed extract (EFLA 940) on BPH related symptoms. During the 12-week intervention, significant effects were reported for IPSS overall and each single question, QoL, and nocturia. Benefits were also documented for the postvoid volume. Different instruments were used in the study, and data consistently confirm the effects on BPH related symptoms, which are beyond expected placebo effects reported from literature. From the data assessed, the oil-free hydroethanolic pumpkin seed extract seems to be an appropriate plant extract to support health benefits in a collective suffering from BPH related symptoms without the need for medical treatment.
